# Supplementary material for: Glycolipid-dependent and lectin-driven transcytosis in mouse enterocytes
Source: Commun Biol. 2021 Feb 9;4:173. doi: 10.1038/s42003-021-01693-2 (PMC7873212; doi:10.1038/s42003-021-01693-2)
Supplement: Supplementary file 2 — Supplementary Information [file 42003_2021_1693_MOESM2_ESM.pdf]

Supplementary Fig. 1: Gal3 and LTF transcytotic uptake is predominantly clathrin-independent

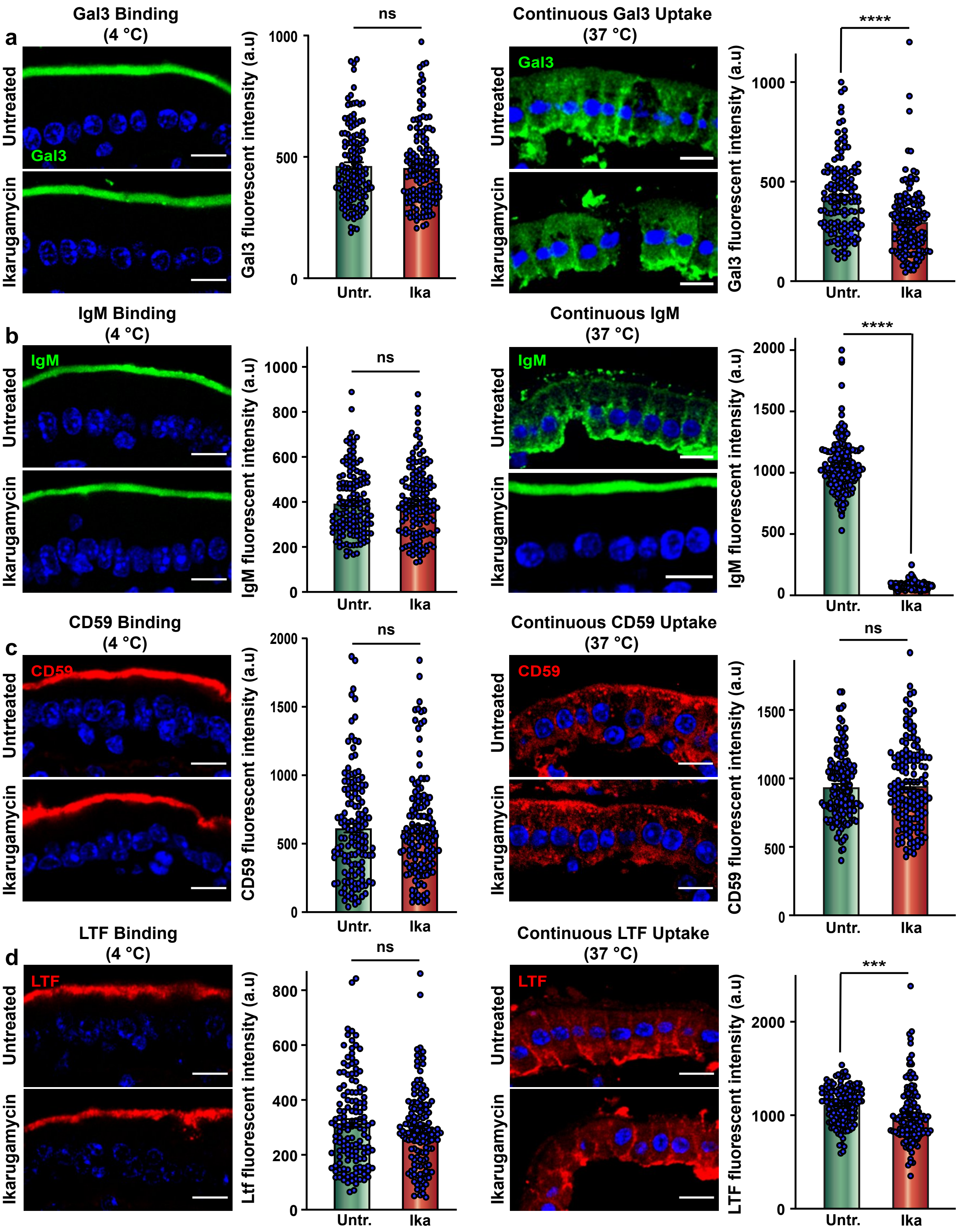

(**a**) DTT-permeabilized jejunum was pre-treated for 30 min with 2 µM Ika prior to incubation in the continuous presence of Ika with 20 µg/ml of Gal3 at 4 °C (left), or at 37 °C (right). Note that Gal3 binding signal at the apical surface (4 °C, left) is similar in both untreated and Ika-treated conditions, while it slightly decreases in Ika conditions when incubation was done at 37 °C (right) (means ± SEM; 4 °C condition: n = 138 cells, 1 representative of 3 independent experiments; 37 °C condition: n = 137 cells, 1 representative of 3 independent experiments). (**b**) Experiment as in (a), except that the incubations were performed with 50 µg/ml of IgM. Although the binding of IgM was the same in untreated and Ika-treated conditions, its internalization consistently dropped to background levels in the presence of Ika (means ± SEM; 4 °C condition: n = 138 cells, 1 representative of 3 independent experiments; 37 °C condition: n = 137 cells, 1 representative of 3 independent experiments). (**c**) Experiment as in (a), except that the incubations were performed with 50 µg/ml of antibody against the GPI-anchored protein CD59 antibody. Note that both binding and endocytosis of anti-CD59 antibody were not altered in the presence of Ika (means ± SEM; 4 °C condition: n = 138 cells, 1 representative of 3 independent experiments; 37 °C condition: n = 137 cells, 1 representative of 3 independent experiments). (**d**) Experiment as in (a), except that the incubations were performed with 20 µg/ml of LTF-Cy3. Similarly to Gal3, LTF binding was not significantly altered by incubation with Ika, and its internalization was only weakly decreased (means ± SEM; 4 °C condition: n = 138 cells, 1 representative of 3 independent experiments; 37 °C condition: n = 137 cells, 1 representative of 3 independent experiments). Nuclei in blue. Student's unpaired t-test, **** p<0.0001, *** p<0.001, ns: non-significant. Scale bars = 10 µm.

Supplementary Fig. 2: GSLs are not required for clathrin-dependent endocytosis

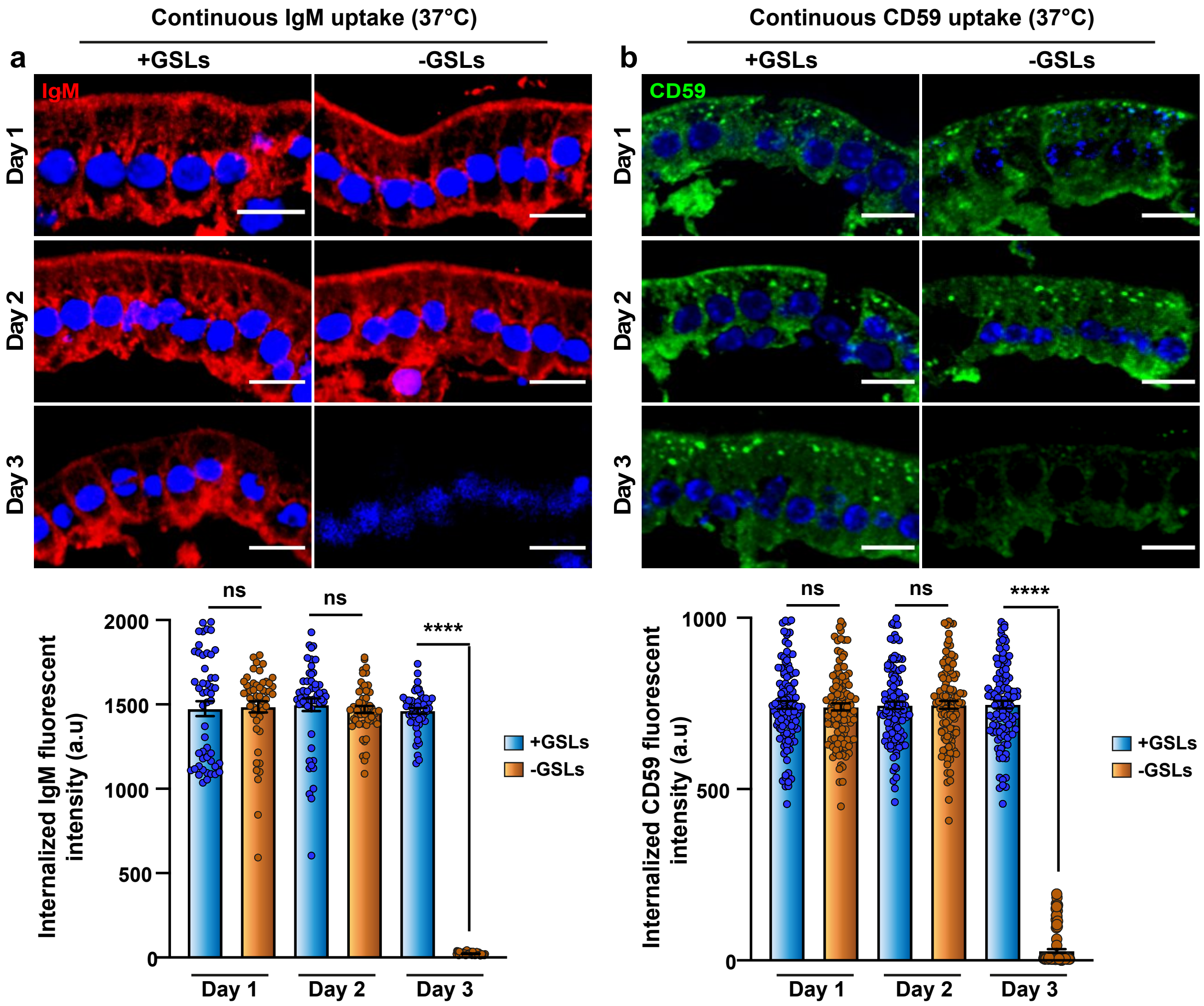

(**a**) DTT-treated jejunum of TAM-injected Ugcgflox/Cre+ (-GSLs) or Ugcgflox/Cre- (+GLSs) mice was continuously incubated at 37 °C with 50 µg/ml of IgM (red). Note that IgM signal was similar at days 1 and 2 after TAM-injection (-GSLs), while it dropped to background levels at day 3 (-GSLs) (means ± SEM, n = 50 cells, 1 representative of 3 independent experiments). (**b**) Experiments as in (a), except that the incubations were performed with 50 µg/ml of anti-CD59 antibody (green). Anti-CD59 endocytosis was not significantly perturbed at day 1 and 2 post-TAM injection (-GSLs), but dramatically dropped to background levels at day 3 (-GSLs) (means ± SEM, n = 109 cells, 1 representative of 3 independent experiments). Nuclei in blue. Student's unpaired t-test, **** p<0.0001, ns: non-significant. Scale bars = 10 µm.

Supplementary Fig. 3: Unprocessed SDS-PAGE of LTF-His/Gal3-Cy3 pull-down experiment

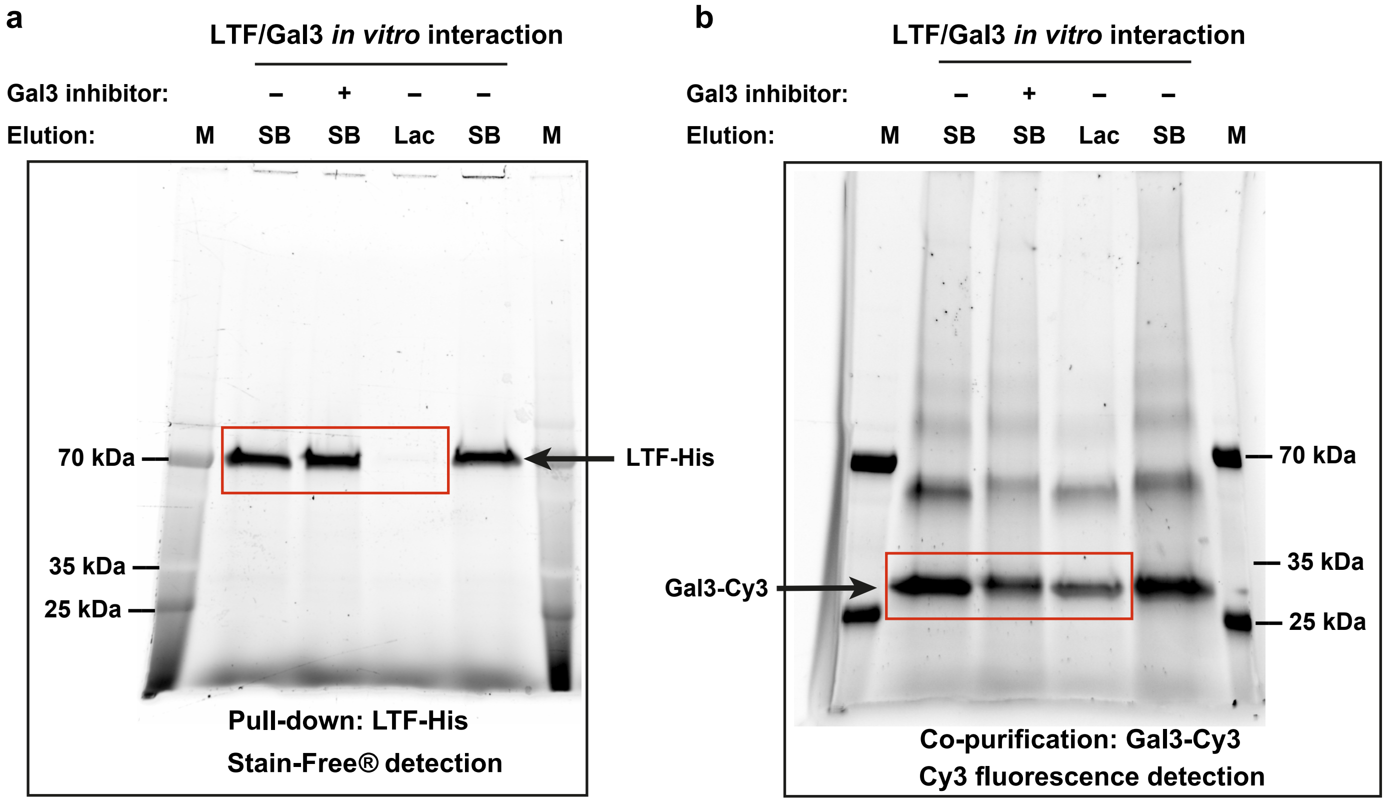

Direct interaction *in vitro of* LTF and Gal3. (**a**) Pull-down experiment on cobalt-agarose beads of purified LTF-His and Gal3-Cy3 in the presence or absence of Gal3 inhibitor. Samples were either eluted with sample buffer (SB) or with 200 mM lactose (Lac), and loaded and run on a Stain-Free® SDS-PAGE gel. Pulled-down LTF (70 kDa band) was detected after UV activation. (**b**) Co-purified Gal3 (around 30 kDa band) was detected using the Cy3 fluorescence filter. Note that Gal3 interacts with LTF in a carbohydrate-dpendent manner. M = PageRuler™ prestained protein ladder.
