# Supplementary material for: Glycolipid-dependent and lectin-driven transcytosis in mouse enterocytes
Source: Commun Biol. 2021 Feb 9;4:173. doi: 10.1038/s42003-021-01693-2 (PMC7873212; doi:10.1038/s42003-021-01693-2)
Supplement: Supplementary file 3 — Reporting Summary [file 42003_2021_1693_MOESM3_ESM.pdf]

nature research

Corresponding author(s): Massiullah Shafaq-Zadah and Ludger Johannes

Last updated by author(s): Dec 8, 2020

Reporting Summary

Nature Research wishes to improve the reproducibility of the work that we publish. This form provides structure for consistency and transparency in reporting. For further information on Nature Research policies, see our Editorial Policies and the Editorial Policy Checklist.

Statistics

For all statistical analyses, confirm that the following items are present in the figure legend, table legend, main text, or Methods section.

n/a	Confirmed	
☐	☒	The exact sample size (*n*) for each experimental group/condition, given as a discrete number and unit of measurement
☐	☒	A statement on whether measurements were taken from distinct samples or whether the same sample was measured repeatedly
☐	☒	The statistical test(s) used AND whether they are one- or two-sided<br>*Only common tests should be described solely by name; describe more complex techniques in the Methods section.*
☒	☐	A description of all covariates tested
☒	☐	A description of any assumptions or corrections, such as tests of normality and adjustment for multiple comparisons
☐	☒	A full description of the statistical parameters including central tendency (e.g. means) or other basic estimates (e.g. regression coefficient) AND variation (e.g. standard deviation) or associated estimates of uncertainty (e.g. confidence intervals)
☒	☐	For null hypothesis testing, the test statistic (e.g. *F*, *t*, *r*) with confidence intervals, effect sizes, degrees of freedom and *P* value noted<br>*Give P values as exact values whenever suitable.*
☒	☐	For Bayesian analysis, information on the choice of priors and Markov chain Monte Carlo settings
☒	☐	For hierarchical and complex designs, identification of the appropriate level for tests and full reporting of outcomes
☒	☐	Estimates of effect sizes (e.g. Cohen's *d*, Pearson's *r*), indicating how they were calculated

Our web collection on statistics for biologists contains articles on many of the points above.

Software and code

Policy information about availability of computer code

Data collection	Nikon A1R confocal microscope from the Nikon Imaging Center was used for fluorescence imaging data collection and Tecnai Spirit 80 KV for electron microscopy
Data analysis	Graphpad Prism 8, version 8.4.3 was used for data analysis. Statistical analysis was performed using unpaired Student's t-test or One-Way ANOVA (for multiple comparison)

For manuscripts utilizing custom algorithms or software that are central to the research but not yet described in published literature, software must be made available to editors and reviewers. We strongly encourage code deposition in a community repository (e.g. GitHub). See the Nature Research guidelines for submitting code & software for further information.

Data

Policy information about availability of data

All manuscripts must include a data availability statement. This statement should provide the following information, where applicable:

- Accession codes, unique identifiers, or web links for publicly available datasets
- A list of figures that have associated raw data
- A description of any restrictions on data availability

The authors declare that all data supporting the findings of this study are available within the paper and its supplementary information files. All MS proteomics data have been deposited to the ProteomeXchange Consortium via the PRIDE partner repository70 with the dataset identifier PXD016499 (username: reviewer18055@ebi.ac.uk, password: ARHPfQ1I). All source data underlying the graphs presented in the main and supplementary figures are available via the following Figshare repository link: https://ndownloader.figshare.com/files/25713287. All other data generated and/or analyzed during the current study are available from the corresponding author upon reasonable request.

Field-specific reporting

Please select the one below that is the best fit for your research. If you are not sure, read the appropriate sections before making your selection.

- [x] Life sciences
- [ ] Behavioural & social sciences
- [ ] Ecological, evolutionary & environmental sciences

For a reference copy of the document with all sections, see nature.com/documents/nr-reporting-summary-flat.pdf

Life sciences study design

All studies must disclose on these points even when the disclosure is negative.

Sample size	No statistical method was used to predetermine sample size. At least 50 cells per condition, collected from different enterocyte cryosections and different mice, were analized to provide a global unbiased quantifications. The precise numbers of cells used per conditions are documented in the figure legends. Experiments were performed at least in triplicates (except when indicated otherwise).
Data exclusions	Using mass spectrometry lipidomic analysis, we have quantified the expression level of GA1 and GlcCer glycosphingolipids (GSLs) species after the genetic removal of the UGCG enzyme, primarily controlling GSLs biosynthesis. This has been performed in the inducible Ugcgflox/Cre-villin mouse model provided by the group of Hermann-Joseph Gröne (ref. 21, "Jennemann, R. et al. Glycosphingolipids are essential for intestinal endocytic function. J. Biol. Chem. 287, 32598-32616, doi:10.1074/jbc.M112.371005 (2012))". It has been well reported (ref. 21) that the expression level of these GSLs species (GA1 and GlcCer) is efficiently down-regulated upon Cre-activation within the Ugcgflox/Cre-villin mice. However, after having performed this lipidomic analysis in this current study, we have indeed found a consistent expression decrease of both GSL-species in 3 replicates out of 4 independent experiments, and an unexpected increase in 1 replicate. We have thereby decided to exclude this aberrant data.
Replication	Each experiment presented in this work was successfully replicated. All experiments have been done at least in triplicates from multiple samples (except when indicated otherwise).
Randomization	No randomization of the sampling was applied in our study.
Blinding	Some experiments have been realized by blinded investigator during data collection

Reporting for specific materials, systems and methods

We require information from authors about some types of materials, experimental systems and methods used in many studies. Here, indicate whether each material, system or method listed is relevant to your study. If you are not sure if a list item applies to your research, read the appropriate section before selecting a response.

Materials & experimental systems

n/a	Involved in the study	
[ ]	[x]	Antibodies
[x]	[ ]	Eukaryotic cell lines
[x]	[ ]	Palaeontology and archaeology
[ ]	[x]	Animals and other organisms
[x]	[ ]	Human research participants
[x]	[ ]	Clinical data
[x]	[ ]	Dual use research of concern

Methods

n/a	Involved in the study	
[x]	[ ]	ChIP-seq
[x]	[ ]	Flow cytometry
[x]	[ ]	MRI-based neuroimaging

Antibodies

Antibodies used	Anti-Gal3 antibody (Fu-Tong Gal Liu, UC Davis, USA), anti-ZO-1 antibody (Abcam, Ref. ab59720, discontinued; Thermo Fisher, Ref. 61-7300), anti-villin antibody (Fatima El Marjou, Institut Curie, France), anti-UEA-1 antibody (Vector Laboratories), anti-CD59 antibody (H-7, sc 133170, Santa Cruz biotechnology, Inc, USA), anti-CD77 antibody (IgM, Joelle Wiels, Institut Gustave Roussy, France)
Validation	All antibodies were quality-controlled and validated by the suppliers and confirmed in-house by immunohistochemistry or flow cytometry. For anti-Gal3 antibody, we used the Gal3 KO mice to validate its specificity. The other antibodies (ZO-1, Villin, UEA-1,...) are broadly used, and their immunofluorescence labeling patterns well described in the literature. The reported labeling patterns of each antibody were perfectly consistent with our sample observations.

Animals and other organisms

Policy information about studies involving animals; ARRIVE guidelines recommended for reporting animal research

Laboratory animals	Details on the mice used in this study are described in the "Methods" section. Mice were of male gender in the age range of 3-4

	months. All animals were housed in a climate-controlled and pathogen-free facility with access to food and water, under a 12 hours light/dark cycle.
Wild animals	This study did not involve wild animals.
Field-collected samples	This study did not involve field-collected samples.
Ethics oversight	Approval of animal experiments and animal handling in a specific pathogen-free animal house facility strictly respected French regulations for animal care. "Comité d'éthique en expérimentation animale de l'institut Curie (CEEA-IC)". National registration number: #118.

Note that full information on the approval of the study protocol must also be provided in the manuscript.
